# The accuracy of point-of-care C-Reactive Protein as a screening test for tuberculosis in children

**DOI:** 10.1371/journal.pgph.0003725

**Published:** 2024-10-24

**Authors:** Mary Kagujje, Sarah Nyangu, Minyoi M. Maimbolwa, Brian Shuma, Nsala Sanjase, Chalilwe Chungu, Andrew D. Kerkhoff, Jacob Creswell, Monde Muyoyeta

**Affiliations:** 1 Tuberculosis Department, Centre of Infectious Disease Research in Zambia (CIDRZ), Lusaka, Zambia; 2 Zambia Paediatric Association, Lusaka, Zambia; 3 Division of HIV, Infectious Diseases and Global Medicine, Zuckerberg San Francisco General Hospital and Trauma Center, University of California San Francisco, San Francisco, California, United States of America; 4 Innovations and Grants, Stop TB Partnership, Geneva, Switzerland; Centro de Investigacao e Treino em Saude da Polana Canico, Instituto Nacional de Saude, MOZAMBIQUE

## Abstract

Systematic screening for TB in children, especially among those at high risk of TB, can promote early diagnosis and treatment of TB. The World Health Organization (WHO) recently recommended C-Reactive Protein as a TB screening tool in adults and adolescents living with HIV (PLHIV). Thus, we aimed to assess the performance of point-of-care (POC) CRP as a screening tool for TB in children. A cross-sectional study was conducted at 2 primary health care facilities in Lusaka, Zambia between September 2020 –August 2021. Consecutive children (aged 5–14 years) presenting for TB services were enrolled irrespective of TB symptoms. All participants were screened for the presence of TB symptoms and signs, asked about TB contact history, and undertook a POC CRP test, chest X-ray, and sputum Xpert MTB/RIF Ultra test. The accuracy of CRP (≥10 mg/L cutoff) was determined using a microbiological reference standard (MRS) and a composite reference standard (CRS). Of 280 children enrolled and with complete results available, the median age was 10 years (IQR 7–12), 56 (20.0%) were HIV positive, 228 (81.4%) had a positive WHO symptom screen for TB, 62 (22.1%) had a close TB contact, and 79 (28.2%) had a positive CRP POC test. Five (1.8%) participants had confirmed TB, 71 (25.4%) had unconfirmed TB, and 204 (72.3%) had unlikely TB. When the MRS was used, the sensitivity of CRP was 80.0% (95%CI: 28.4–99.5%) and the specificity was 72.7% (95%CI: 67.1–77.9%). When the CRS was used, the sensitivity of CRP was 32.0% (95%CI: 23.3% - 42.5%), while the specificity was 74.0% (95%CI: 67.0% - 80.3%). Using the CRS, there were no statistically significant differences in sensitivity and specificity of CRP in the HIV positive and HIV negative individuals. Among children in Zambia, POC CRP had limited utility as a screening tool for TB. There remains a continued urgent need for better tools and strategies to improve TB detection in children.

## Background

In 2022, the global burden of TB was estimated to be 10.6 million people, of whom 1.1 million were children less than 15 years old [1]. That same year, only about 55% of children who became ill with TB were diagnosed and reported to national TB programmes. Furthermore, TB resulted in approximately 213,000 deaths among children (contributing 17% and 19% of mortality among the HIV negative and the HIV positive respectively). Notably, over 90% of the children who die from TB were not receiving TB treatment at the time of their death [2], underscoring the substantial barriers to accessing timely diagnosis and treatment for this age group. In Zambia, it is estimated that 8,800 children less than 15 years of age developed TB in 2022, yet only 5,953 (68%) children were notified.

The World Health Organization (WHO) recommends systematic screening for TB in children who are at increased risk of TB especially those living with HIV and close contacts to persons with TB [3]. This strategy not only promotes early diagnosis and treatment but also uptake of TB preventive treatment in eligible children, mitigating TB’s impact within this vulnerable age-group. The current, WHO-recommended TB screening approaches for children under 15 years of age are symptom-based screening and/or chest x-ray (CXR) [3]. However, findings from a recent systematic review highlights the limitations of symptom-based screening strategies [4]. For instance, a symptoms-based screening approach used in four studies only approximated the WHO target product profile (TPP [90% sensitivity and 70% specificity] [5]) among household contacts; the accuracy of other symptoms-based approaches was sub-optimal for other high-risk groups [4]. Further, although CXR typically outperforms symptom screening [3], they are often inaccessible in most resource-limited, high-TB burden settings, particularly at lower healthcare setting levels, where children usually first present.

The WHO has recently endorsed C-Reactive Protein (CRP) as a TB screening tool in adults and adolescents living with HIV. As an inflammatory biomarker, CRP is useful in ruling out active TB disease, and monitoring response to treatment [6]. It demonstrates similar or higher sensitivity compared to the symptom screening [3], and is available as a low-cost, point-of-care (POC) tool, that can be utilized at any level of the health care system. However, literature on the utility of CRP for TB screening in children is limited reporting a sensitivity and specificity ranging between 38%-56% and 54%-65% respectively [7]. Furthermore, given that children with TB typically exhibit smaller increases in CRP concentrations than adults with TB [8], the performance of CRP cannot be extrapolated from adults to children. Therefore, this study aimed to evaluate the accuracy of CRP as a screening tool for TB in children.

## Methods

### Study participants

Between 10^th^ September 2020 and 11^th^ August 2021, we recruited participants under a prospective cross-sectional study at two primary health care facilities in Lusaka, Zambia. The study sample size was 300 which was determined using the following assumptions: expected sensitivity and specificity of 0.86 and 0. 35 respectively [9], prevalence of 0.16 in the population and 3.5% of the recruited children failing to submit samples. We consecutively enrolled children aged 5–14, who presented for TB screening at the fast-track TB services desk that was stationed at the health facility or that were identified through contact tracing under the TB REACH study [10], regardless of whether they showed TB symptoms at presentation. These children were identified through a TB case finding study that was enhancing contact tracing and also providing fast track TB services at the health facility [10]. The fast track desk attended both to children that were brought in by their caregivers following the health talks and those referred by health care workers and community health workers from all health facility departments, especially the pediatric anti-retroviral therapy (ART) clinic, nutritional department, and outpatient department (OPD) [10].

The study was approved by the University of Zambia Biomedical Research Ethics Committee (Ref No: 635–2020) and National Health Research Authority. For participants below the age of seven, we obtained written informed consent from their guardians. For those between 7–14 years old, we secured written informed assent, with the child’s guardian signing as a witness. Confidentiality was maintained, and the study database did not contain any personal identifiers. This study conforms to the Standards for Reporting of Diagnostic Accuracy Studies (STARD) initiative guidelines [11].

### Procedures

Trained study personnel collected participants’ demographic and clinical data at the time of enrolment, including the WHO-recommended four-symptom screen (any current cough, fever, weight loss and night sweats of any duration), contact history to a person with bacteriologically confirmed TB, vital signs, and physical examination findings. A semi-quantitative POC CRP test, Actim Rapid CRP kit (Oy Medix Biochemica Ab, Finland) [12], with three thresholds corresponding to 10, 40 and 80 mg/mL was used. The test was performed at enrolment using a finger stick blood sample. A study clinician trained and experienced in this procure carried out the test according to the kit’s standard operating procedure.

Under the guidance of an experienced research assistant, a spot sputum sample was collected from all participants able to spontaneously produce sputum. For those unable to expectorate a sample, a trained healthcare worker performed sputum induction. Sputum samples were tested for TB using the Xpert MTB/RIF Ultra assay (Cepheid, USA) at the local facility laboratory within 24 hours of collection using standard protocols. Tests with errors or invalid results were repeated.

Xpert MTB/RIF Ultra was used as the microbiological reference standard due to its ready availability in our setting as well as its considerably high performance in diagnosing TB in children [13]. The clinical team did not have access to the Xpert MTB/RIF Ultra results while performing the CRP test.

Children also received a digital CXR, using an ultra-portable digital Fujifilm X-ray machine (Fujifilm, Japan). The x-ray images were interpreted by three radiologists. CXRs were read independently by each expert reader who scored each x-ray using a standard form. The final conclusion on each image was abnormal TB, Abnormal not TB and Normal. Agreement by at least 2 readers was considered as a final result.

HIV testing was conducted using the Alere Determine HIV-1/2 test (AlereHIV-1/2; Abbott, Chicago, Illinois, USA). Positive results were confirmed using the SD Bioline HIV-1/2 (SD Bioline HIV-1/2; Abbott). If a child was known to be HIV positive or had a documented HIV negative result within the past 3 months, no repeat testing was done.

### Definitions

We established a priori that a POC CRP concentration of ≥10 mg/L would be considered a positive screen for TB, based on the manufacturer’s instructions [12]. In accordance with WHO recommendations, individuals reporting any one of the four TB symptoms (any duration) were deemed symptom screen positive. An Xpert MTB/RIF Ultra trace call result was interpreted as positive in all children. A chest x-ray was defined as abnormal if it had an any chest x-ray abnormality or abnormalities consistent with active TB disease.

Upon evaluating participants’ clinical, radiological and laboratory data, we classified them into one of three mutually exclusive groups using the updated clinical criteria for pediatric TB for research studies: (1) Confirmed TB (at least one symptom or sign of TB AND a positive Xpert MTB/RIF Ultra result on sputum); (2) Unconfirmed TB (no bacteriological confirmation AND at least two of the following–(a) current symptoms/signs suggestive TB, (b) a chest x-ray consistent with TB and (c) close contact to a confirmed TB case); and Unlikely TB (no bacteriological confirmation AND criteria for unconfirmed TB not met) [14, 15].

We used two reference standards, the microbiological reference standard (MRS) and the composite reference standard (CRS). When using the MRS, TB-positive was defined as a child meeting the definition of “confirmed TB,” while TB-negative was defined as a child classified as “unconfirmed TB” or “unlikely TB.” When CRS, TB positive was defined as a child meeting the definition of "confirmed TB” or “unconfirmed TB” a TB negative was defined as a child classified as “unlikely TB”.

### Statistical analysis

All statistical analyses were performed using STATA version 18 (Stata Corp LLC). Participants who were missing data on CRP or Xpert MTB/Rif Ultra (included those with results reporting error on repeat testing) were excluded from the analysis. Z-scores were generated using Body Mass Index (BMI) and the WHO reference standard BMI for age. To characterize the study population, we conducted a descriptive analysis. Categorical variables are presented as numbers and percentages, while continuous variables are reported as medians and interquartile ranges.

We calculated the point estimates and 95% CIs for the sensitivity, specificity, negative predictive value (NPV), and positive predictive value (PPV) of CRP using a both the MRS and the CRS. Both analyses were stratified by HIV status in order to further understand any differences between the HIV and non-HIV groups. Further, we determined the area under the receiver operator curve (AUROC) for CRP against both the MRS and CRS.

## Results

### Study population

**Fig 1:**
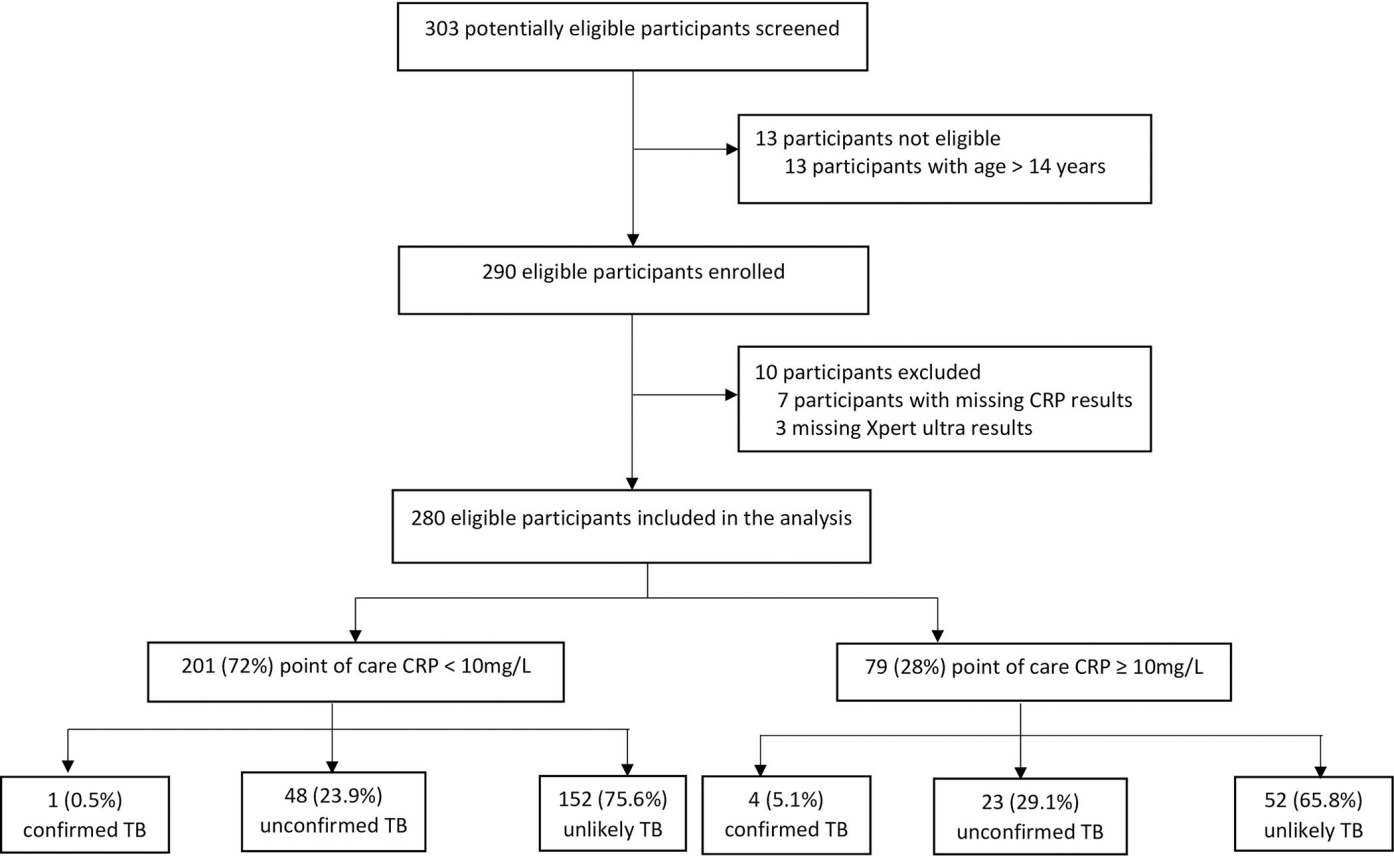
Study flow diagram.

A total of 303 individuals were approached to participate in the study of whom 290 were eligible and were enrolled (**Fig 1**). Of these, 280 (96.6%) participants had complete CRP and Xpert MTB/RIF Ultra results and were included in the analysis.

The median age for the study participants was 10 years old (IQR 7–11), 141 (50.4%) were male, and 56 (20.0%) were HIV positive (**Table 1**). There were 228 (81.4%) participants who had at least one symptom of TB (according to the WHO symptom screen), 62 (22.1%) had a close contact history with a person with confirmed TB, 37 (13.2%) had an abnormal CXR consistent with TB and 122 (43.6%) that were malnourished. Among individuals who tested positive for C-reactive protein (CRP), a majority (73.4%), had CRP levels within the range of 10–40 mg/L (Table 1). Among children diagnosed with confirmed TB, 40.0% had a CRP value in the range of >80mg/L. Among children diagnosed with TB (confirmed and unconfirmed TB), majority (64.4%) had a CRP value in the range of <10 mg/L while among those with TB unlikely, majority (74.5%) had a CRP value in the range of 10 mg/L.

**Table 1:** Demographic and clinical characteristics of participants.

Characteristic	All children (n = 280)	CONFIRMED TB (n = 5)	UNCONFIRMED TB (n = 71)	UNLIKELY TB (n = 204)
MEDIAN AGE, (IQR) YEARS	10 (7–12)	10 (7–12)	9 (6–11)	10 (7–12)
MALE	141 (50.4)	0 (0.0)	34 (47.9)	107 (52.4)
HIV POSITIVE	56 (20.0)	1 (20.0)	9 (12.7)	46 (33.6)
CLOSE TB CONTACT	62 (22.1)	0 (0.0)	54 (87.9)	8 (3.9)
COUGH	210 (75.3)	5 (100)	62 (87.3)	143 (70.1)
FEVER	105 (37.5)	3 (60.0)	38 (53.5)	64 (31.4)
NIGHT SWEATS	46 (16.4)	2 (40.0)	24 (33.8)	20 (9.8)
WEIGHT LOSS	116 (41.4)	3(60.0)	44 (62.0)	69 (33.8)
WHO SYMPTOM SCREEN	228 (81.4)	5 (100)	71 (100)	152 (74.5)
CRP POSITIVE^#^	79 (28.2)	4 (80.0)	23 (32.4)	52 (25.5)
CRP RANGE				
<10mg/L	201 (71.8)	1 (20.0)	48 (67.6)	152 (74.5)
10–39.9mg/L	58 (20.7)	1 (20.0)	13 (18.3)	44 (21.6)
40-80mg/L	8 (2.9)	1 (20.0)	2 (2.8)	5 (2.5)
>80mg/L	13 (4.6)	2 (40.0)	8 (11.3)	3 (2.4)
ABNORMAL CXR^^^	37 (13.2)	4 (80.0)	15 (21.1)	0 (0.0)
**Z-SCORE**				
**NORMAL**	55 (19.4)	0	14 (19.7)	41 (20.1)
**-1SD**	53 (18.9)	2(40.0)	13 (18.3)	38 (18.6)
**-2SD**	29 (10.4)	1 (20.0)	6 (8.5)	22 (10.8)
**-3SD**	40 (14.3)	1(20.0)	10 (14.1)	29 (14.2)
**+1SD**	40 (14.3)	0	15 (21.3)	25 (12.3)
**+2SD**	18(6.4)	0	3 (4.2)	15 (7.4)
**+3SD**	22 (7.9)	0	4 (5.6)	18 (8.8)

*All values represent n (%) except where explicitly noted.

^#^CRP positive defined by a value ≥ 10mg/L.

^^^The presence of any chest X-ray abnormality(ies) consistent with active TB disease.

IQR = Interquartile range, HIV = Human Immunodeficiency virus, TB = Tuberculosis, CRP = C-reactive protein, WHO = World Health Organisation and CXR = Chest x-ray

### Accuracy of CRP for TB

The sensitivity, specificity, PPV, and NPV of POC CRP for TB are shown in **Table** 2. Against a MRS, the sensitivity and specificity of CRP was 80.0% and 72.7% respectively, while the NPV and PPV was 99.5% and 5.1%, respectively. The small number of TB cases precluded the ability to evaluate accuracy according to HIV status.

**Table 2:** Accuracy of point-of-care C-reactive protein for tuberculosis among children according to microbiological (MRS) and composite reference standards (CRS).

	Accuracy using MRS	Accuracy using CRS
Sensitivity	80.0% (28.4%-99.5%)	35.5% (24.9–47.3)
Specificity	72.7% (67.1%-77.9%)	74.5% (68.0–80.3)
NPV	99.5% (97.3%-100%)	75.6% (69.1–81.4)
PPV	5.1% (1.4%-12.5%)	34.2% (23.9–45.7)

MRS = Microbiological Reference standard, CRS = Composite Reference Standard, NPV = Negative Predictive Value, PPV = Positive Predictive Value

**Fig 2:**
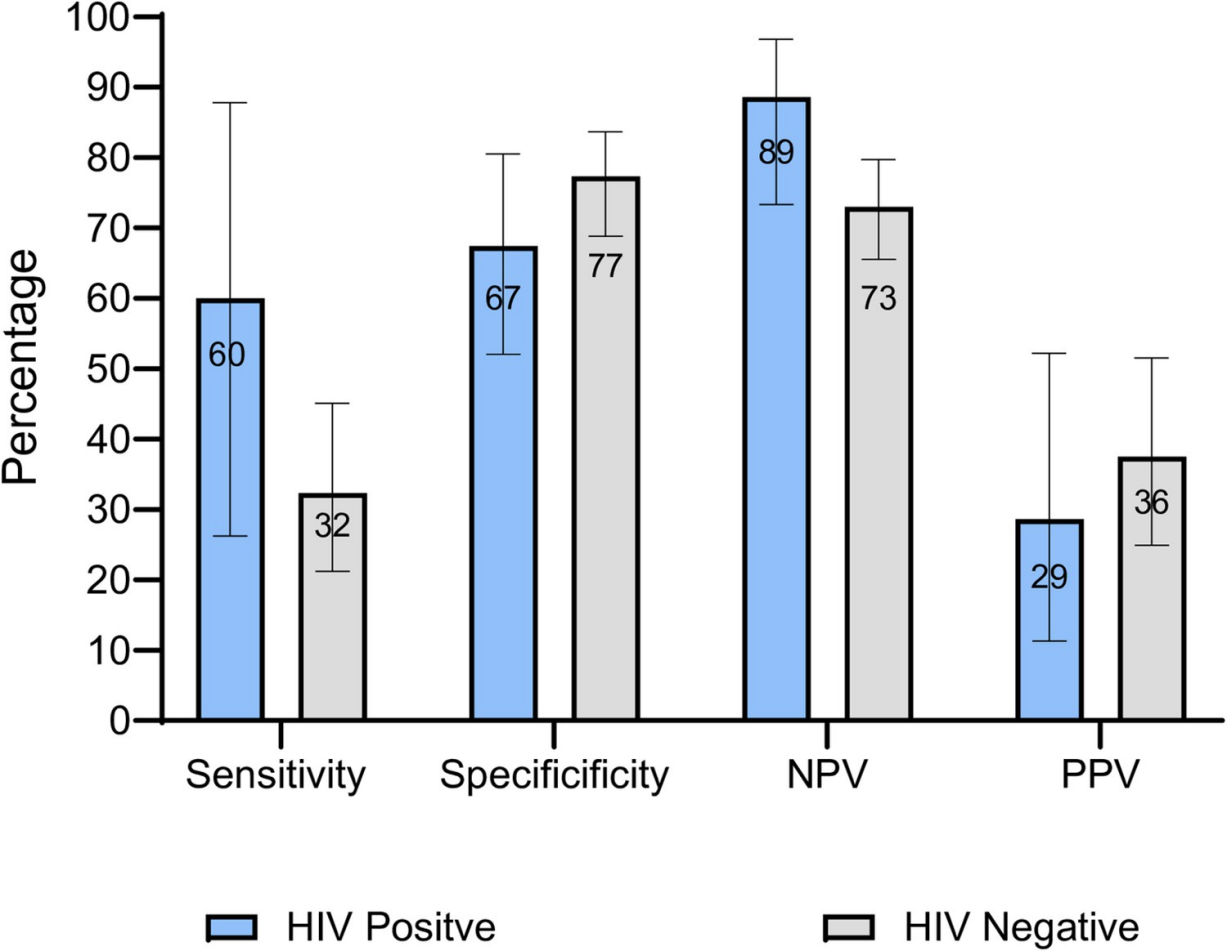
Accuracy of point-of-care C-reactive protein for tuberculosis screening among children according to HIV status, using a composite reference standard (CRS).

When the CRS definition was used, among all children the sensitivity and specificity of POC CRP was 35.5% and 74.5%, respectively, while the NPV and PPV were 75.6% and 34.2%, respectively. The accuracy of POC CRP according to HIV status, using the CRS, is shown in **Fig 2.** There was a trend toward higher sensitivity (60.0% vs. 32.3%) and lower specificity (67.4% vs. 77.3%) among HIV positive children compared to HIV negative children, however, confidence intervals were overlapping.

**Fig 3:**
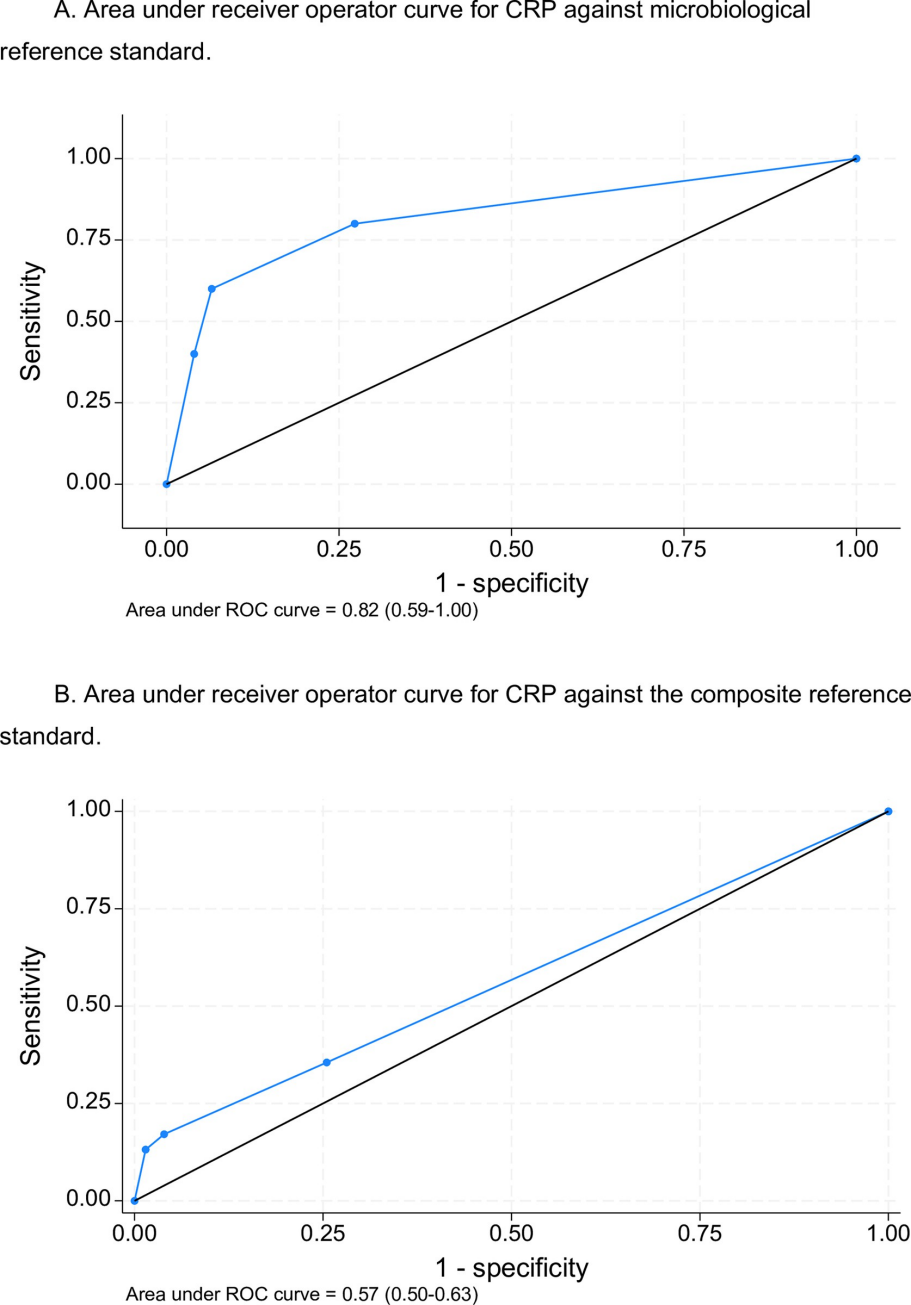
A. Area under receiver operator curve for CRP against microbiological reference standard. B. Area under receiver operator curve for CRP against the composite reference standard.

CRP had considerable discriminatory value when the MRS was used AUC 0.82(0.59–1.0) (Fig 3A) while it failed to discriminate between TB and no TB when the CRS was used AUC 0.57 (0.50–0.63) (Fig 3B).

## Discussion

In this study among HIV-positive and negative children presenting to public health facilities in Lusaka, Zambia, POC CRP demonstrated limited utility as a standalone screening tool for pulmonary TB. While CRP had good sensitivity and excellent NPV when utilizing a MRS, it did not achieve the target sensitivity of 90% for new TB screening tools recommended in WHO’s TPP. Additionally, when using the MRS, CRP demonstrates considerable performance in discriminating those with TB from those without TB. However, given the limitations of existing diagnostic tests for bacteriologically confirming TB in children, the MRS very likely overestimates the true discriminatory value, sensitivity and NPV of CRP for TB. We didn’t stratify the accuracy of CRP by HIV status, against the MRS, due to the limited number of children with microbiologically confirmed TB. When a CRS definition was applied, the sensitivity and NPV of CRP was only low-to-moderate, irrespective of HIV status and the AUROC suggests that CRP failed to discriminate those with TB from those without TB. Our results highlight the continued urgent need for the development of improved, low-cost, point-of-care screening tools to improve the detection of TB in children.

Our study aligns with and expands on the extremely limited body of research on the use of CRP for TB screening in children. To our knowledge, no other study has assessed the performance of CRP as a screening tool for TB in children. The only study to-date, assessed the CRP as a triage tool among symptomatic children in Uganda [7]. This study used the same definitions for MRS and CRS to those used by our study and it reports largely similar findings to those reported by our study. In both studies, the sensitivity of CRP among children fell well short of recommended thresholds for TB screening tests. Also, the specificity of CRP appeared to be slightly reduced among HIV positive children, a phenomenon potentially attributable to their heightened risk of other bacterial infections, which could also cause increased CRP levels. Notably, the point estimates for specificity of CRP in the HIV positive children that were reported by our study were much higher than those reported by the Ugandan study (67% vs 27%). However, this did not reach significance as the confidence intervals are overlapping and hence could be attributable to the differences in sample sizes.

Overall, irrespective of the whether the MRS or CRS is used, the point estimates for CRP sensitivity in TB screening are lower (80%/36% vs. 92%) and specificity is higher(73%/75% vs 37%) in children compared to adults [9]. The difference in CRP sensitivity and specificity between children and adults is more pronounced in individuals that are HIV negative [9, 16–19]. In HIV-negative children, CRP sensitivity is less than half of that reported in adults (32% vs. 78%-84%) [9, 19], whereas in HIV-positive children, the sensitivity is lower but the difference is smaller (60% vs. 85%-92%). Additionally, in HIV-negative children, CRP specificity is higher than that in adults (77% vs. 25%-52%) [9, 19], while in HIV-positive children, the specificity varies widely and in some studies is even comparable to that in adults (67% vs. 37%-72%) [16–18]. This lower sensitivity in children is attributed to typically smaller increases in CRP concentrations in children with TB than in adults with TB [7, 8, 20, 21]. This aligns with evidence from our study where majority (64.4%) of the children diagnosed with TB (confirmed and unconfirmed TB) had CRP values <10mg/L as well as the study conducted in Uganda where the median CRP among children diagnosed with TB was <10mg/L [7]. This is in contrast to adults with TB who have CRP values between 15-83mg/L [17, 22, 23]. The higher specificity of CRP in children suggests a lower prevalence of co-morbidities [24–29], that can also lead to raised CRP levels. However, our study didn’t evaluate the participants for the comorbidities associated with CRP increase.

In adults, parallel use of CRP and symptoms led increased the sensitivity to 100% (93.8–100) [9]. In our analysis, we did not explore the parallel use of CRP and symptoms/chest x-ray as our MRS definition includes symptoms and our CRS definition also includes both symptoms and x-ray findings. This overlap would have resulted into an overestimation of sensitivity and specificity.

The strengths of our study include that the paediatric TB diagnosis in our study was clearly defined following international standards [14], and the inclusion of both HIV positive and HIV negative children in our study sample, which allowed for an of assessment of CRP’s performance by HIV-status against the composite reference standard. However, our study is not without limitations. Firstly, the sample size was not powered to detect differences by HIV status. Secondly, our use of a 10 mg/L of CRP may have contributed to a lower sensitivity especially that children with TB typically exhibit smaller increases in CRP concentrations than adults with TB [8]. However in the only other publication evaluating CRP’s performance among children, a 5 mg/L cutoff did not meaningfully improve sensitivity compared to a 10 mg/L cutoff [7]. Thirdly, our study had very low numbers under the MRS causing the wide confidence intervals for sensitivity and also limiting our ability to do a stratified analysis by HIV status using the MRS definition. Consequently, we had a bigger focus on results from the CRS definition. However, one limitation of using the CRS is that some of the children with unconfirmed TB may be falsely classified as having TB which could result in wrong estimates of the accuracy of the test. Fourth, our study focused solely on pulmonary TB and thus did not explore the performance of CRP for detecting extrapulmonary TB. Given that extrapulmonary TB is a relatively common manifestation in children, and may be associated with higher blood CRP levels than isolated pulmonary disease [30], this represents an area for future research. Lastly, we didn’t include children <5 years who are often more difficult to diagnosed with TB. Our results hence can’t be generalised to this group.

In conclusion, CRP as a standalone TB screening test in both HIV positive and negative children has limited value. Consequently, its utility in facilitating systematic TB screening among children is limited. Improving outcomes for this vulnerable population urgently requires the development of novel tools and strategies to enhance TB screening and diagnosis in children.

## Supporting information

S1 Fig Proportion of participants in each CRP categorization. (TIF)

S1 Data Study dataset. (XLSX)
