# Supplementary figures and images for: The accuracy of point-of-care C-Reactive Protein as a screening test for tuberculosis in children
Source: PLOS Glob Public Health. 2024 Oct 24;4(10):e0003725. doi: 10.1371/journal.pgph.0003725 (PMC11500861; doi:10.1371/journal.pgph.0003725)

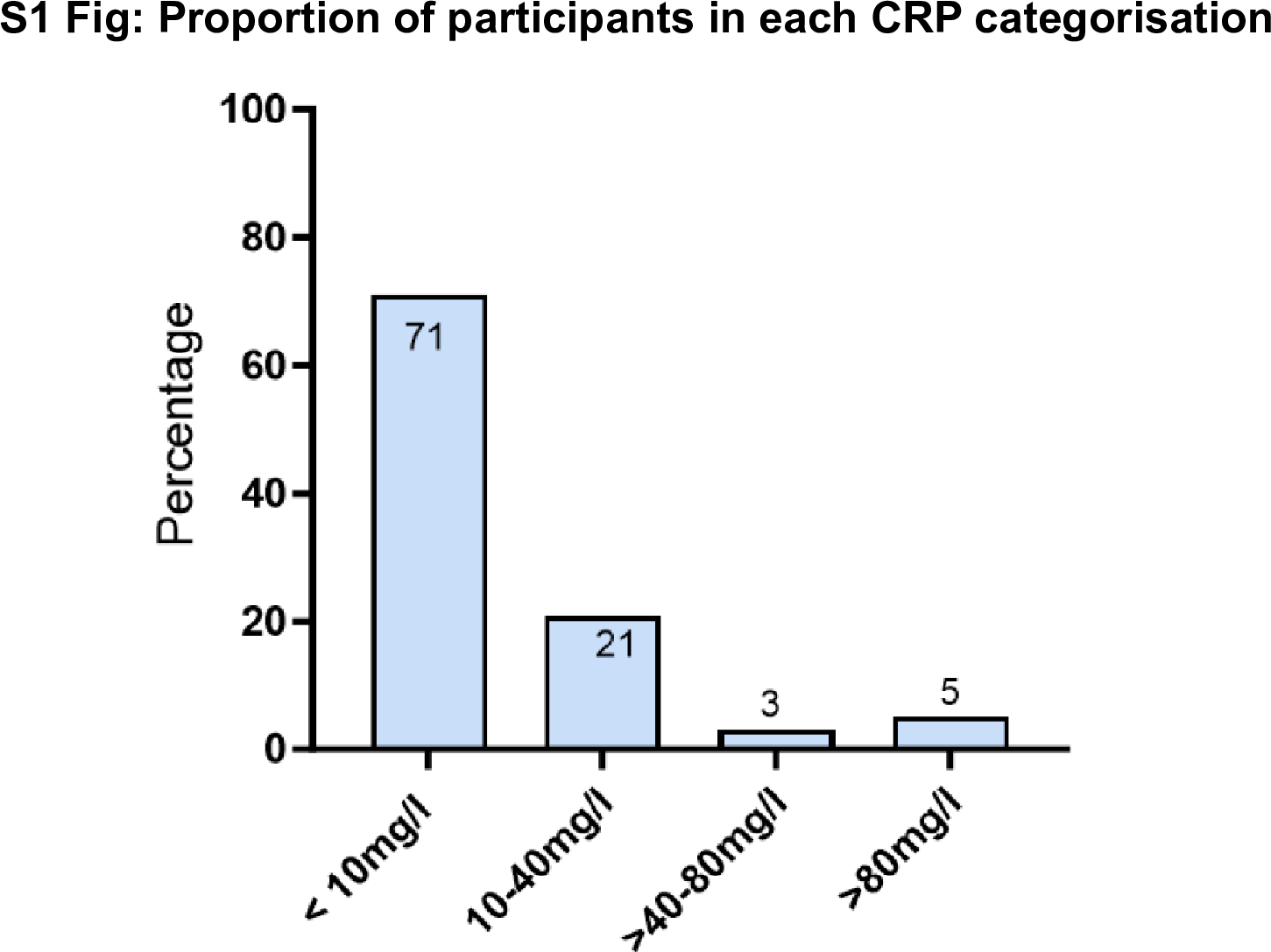

Supplement: S1 Fig — (TIF) [file pgph.0003725.s001.tif]
